# Risk factors associated with cluster size of *Mycobacterium tuberculosis* (Mtb) of different RFLP lineages in Brazil

**DOI:** 10.1186/s12879-018-2969-0

**Published:** 2018-02-08

**Authors:** Renata Lyrio Peres, Solange Alves Vinhas, Fabíola Karla Correa Ribeiro, Moisés Palaci, Thiago Nascimento do Prado, Bárbara Reis-Santos, Eliana Zandonade, Philip Noel Suffys, Jonathan E. Golub, Lee W. Riley, Ethel Leonor Maciel

**Affiliations:** 1 Núcleo de Doenças Infecciosas, Universidade Federal do Espírito Santo, Vitória, Espirito Santo, Brazil; 2 Laboratório de Epidemiologia da Universidade Federal do Espírito Santo, Av. Marechal Campos, 1468- Maruípe-, Vitória, ES Brazil; 3 Programa de Pós-Graduação em Epidemiologia, Universidade Federal de Pelotas, Pelotas, Brazil; 4 Laboratório de Biologia Molecular Aplicada a Micobactérias, Instituto Oswaldo Cruz – FioCruz, Rio de Janeiro, Brazil; 5 School of Medicine, Johns Hopkins University, Baltimore, MD USA; 6 Division of Infectious Disease and Vaccinology, School of Public Health, University of California, Berkeley, CA USA

**Keywords:** Tuberculosis, Molecular epidemiology, Transmission, Risk factors, Cluster size

## Abstract

**Background:**

Tuberculosis (TB) transmission is influenced by patient-related risk, environment and bacteriological factors. We determined the risk factors associated with cluster size of IS*6110* RFLP based genotypes of *Mycobacterium tuberculosis* (Mtb) isolates from Vitoria, Espirito Santo, Brazil.

**Methods:**

Cross-sectional study of new TB cases identified in the metropolitan area of Vitoria, Brazil between 2000 and 2010. Mtb isolates were genotyped by the IS*6110* RFLP, spoligotyping and RD^Rio^. The isolates were classified according to genotype cluster sizes by three genotyping methods and associated patient epidemiologic characteristics. Regression Model was performed to identify factors associated with cluster size.

**Results:**

Among 959 Mtb isolates, 461 (48%) cases had an isolate that belonged to an RFLP cluster, and six clusters with ten or more isolates were identified. Of the isolates spoligotyped, 448 (52%) were classified as LAM and 412 (48%) as non-LAM. Our regression model found that 6–9 isolates/RFLP cluster were more likely belong to the LAM family, having the RD^Rio^ genotype and to be smear-positive (adjusted OR = 1.17, 95% CI 1.08–1.26; adjusted OR = 1.25, 95% CI 1.14–1.37; crude OR = 2.68, 95% IC 1.13–6.34; respectively) and living in a Serra city neighborhood decrease the risk of being in the 6–9 isolates/RFLP cluster (adjusted OR = 0.29, 95% CI, 0.10–0.84), than in the others groups. Individuals aged 21 to 30, 31 to 40 and > 50 years were less likely of belonging the 2–5 isolates/RFLP cluster than unique patterns compared to individuals < 20 years of age (adjusted OR = 0.49, 95% CI 0.28–0.85, OR = 0.43 95% CI 0.24–0.77and OR = 0. 49, 95% CI 0.26–0.91), respectively. The extrapulmonary disease was less likely to occur in those infected with strains in the 2–5 isolates/cluster group (adjustment OR = 0.45, 95% CI 0.24–0.85) than unique patterns.

**Conclusions:**

We found that a large proportion of new TB infections in Vitoria is caused by prevalent Mtb genotypes belonging to the LAM family and RD^Rio^ genotypes. Such information demonstrates that some genotypes are more likely to cause recent transmission. Targeting interventions such as screening in specific areas and social risk groups, should be a priority for reducing transmission.

## Background

Tuberculosis (TB) continues to be a challenge to control. Although widespread and common efforts have had an impact in achieving declining numbers in global incidence for the first time in history, TB still causes 10.4 million new cases and 1.4 million deaths per year in worldwide [1].

Brazil ranks sixteenth among the world’s 22 countries with high TB burdens; here, in 2016, the TB incidence was 63,189 cases, and the incidence rate was 30.9 per 100,000 per year, with mortality rate of 2.2 per 100,000 according to World Health Organization estimates [1]. The state of Espírito Santo has one of the lowest incidence of tuberculosis (28.6 / 100,000 / year) in Brazil [2]. Vitoria is a large urban setting and capital of Espírito Santo state, and reports over 279 cases of TB each year, which is among the highest incidence in the country (40.2/100,000/year) [3].

Understanding how TB transmission occurs is a key component to strategically manage TB from a public health perspective. *Mycobacterium tuberculosis* complex (MTBC) genotyping methods have been widely used in in molecular epidemiological studies [4]. These methods help to detect its spread, understand the dynamics of the disease, and develop tuberculosis (TB) control strategies to minimize TB expansion locally and globally.

Studies have suggested that identical IS6110 RFLP patterns of *M. tuberculosis* (Mtb) isolates from epidemiologically linked patients reflect TB resulting from recent transmission [5, 6]. Subsequent cases in transmission chains result in “clusters” of patients who share Mtb strains of the same genotype [7]. If a large proportion of new TB cases in a given community are due to recent transmissions, this is a reflection of an inadequate TB control program.

Many studies have investigated risk factors for clustering, suggesting that patient-related risk factors are important for TB transmission [5, 6, 8]. There is substantial evidence, however, that bacterial factors also contribute to variability in cluster size and the extent of transmission of TB in a community [8]. Indeed molecular epidemiologic studies have suggested that some strains are more successfully transmitted than others [9–11].

We performed genotyping of a large collection of Mtb strains that had been collected over a 11-year period in the metropolitan area of Vitoria, Espirito Santo state, Brazil and evaluated the relation between genotypes and clustering and strain and epidemiologic, clinical, and demographic characteristics. Our hypothesis was that this approach might help better understand risk factors for recent TB transmission in this particular setting.

## Methods

### Study population

This cross-sectional study examined all TB patients newly diagnosed in the metropolitan area of Vitoria, Brazil between 2000 and 2010. The Metropolitan area comprises four municipalities (Vitória, Cariacica, Serra and Vila Velha) with about 1,200,000 inhabitants. The study sample included isolates from all patients with positive culture results. The isolates were classified according to cluster size of Mtb strains and their associations with molecular and epidemiologic features were assessed.

### Genotyping methods

#### IS*6110* restriction fragment length polymorphism (RFLP) analysis

Sputum cultures for TB diagnosis are done routinely by the reference Mycobacteriology Laboratory at Núcleo de Doenças Infecciosas at the Federal University of Espirito Santo (NDI – UFES). We analyzed all available stored Mtb isolates that were consecutively obtained at reference laboratory for genotype analysis.

We used the standard IS*6110* RFLP protocol [12] to genotype the isolates. Briefly, the genomic mycobacterial DNA was extracted, digested, and separated by gel electrophoresis. The DNA fragments resolved in agarose gel were transferred to a Hybond N-Plus membrane (GE Healthcare Life Sciences) and were hybridized with a probe made from a PCR product of the 3′ part of the *Pvu*II fragment of IS*6110*. The IS*6110* containing fragments on the membrane were detected by chemiluminescence (ECL direct™ nucleic acid labeling and detection system, GE Healthcare Limited, UK) and exposure to an X-ray film (A Hyperfilm™ ECL, GE Healthcare Limited, UK). The Mtb 14,323 strain was used as a reference strain for comparison of the RFLP patterns.

The IS*6110* RFLP band patterns were analyzed by the BioNumerics software version 6.5 (Applied Maths – Belgium). A dendrogram was constructed to show the degree of similarity among the isolates by unweighted pair group method of arithmetic average (UPGMA) and the Dice index (1.0% tolerance, 1.5% optimization).

Two or more isolates with identical RFLP patterns (fingerprint) were defined as belonging to a cluster while strains with RFLP patterns of at least 70% similarity were considered members of the same “family”. As described in other studies, isolates belonging to a cluster were considered to result from recent infections while isolates whose RFLP patterns were distinctly different from any other pattern identified among the isolates studied were considered unique or non-cluster patterns and were considered to represent reactivation from an old infection. Clusters composed patterns with less than six bands were tested by spoligotyping as this increases cluster reliability [13–16]. We named the clusters in our study with an abbreviation of ES, for Espírito Santo State.

##### Spoligotyping

Isolates were also submitted to spoligotyping by a commercial kit (Ocimum Biosolutions Inc., India) according to a standard protocol [17, 18], allowing the classification of strains into spoligotype-based families, based on the presence or absence of spacer regions. Results were recorded in a 43-digit binary format and compared with an updated SpolDB4 [18] database – SITVITWEB [19] of the Pasteur Institute of Guadeloupe (available at http//:www.pasteur-guadeloupe.fr:8081/SITVITDemo/) that provides information on the Mtb spoligotypes worldwide. The orphan patterns were entered into SPOTCLUST [20] in order to define the probability of a strain to belong to a certain family.

##### Long sequence polymorphism (LSP)

A multiplex PCR adapted from Gibson et al. [21] was performed to identify isolates of the RD^Rio^ genotype. The differentiation of RD^Rio^ from non-RD^Rio^ was determined according to the PCR product band size; the presence of a band of 1175 bp indicated RD^Rio^ while a band of 530-bp identified non- RD^Rio^ strains.

### Epidemiological, clinical and molecular characteristics

We obtained general epidemiologic characteristics including gender, age, race, schooling (years), and previous history of TB, from the Brazilian national surveillance system (SINAN) and also from laboratory records maintained at the NDI-UFES. SINAN is the Brazilian Information System for notifiable diseases and its data are publicly accessible via the website of the Data Processing Department of Brazilian Ministry of Health (DATASUS) [22].

The following socio-demographic variables were evaluated: age (< 20 years, 21–30 years, 31–40 years, 41–50 years and > 50 years), gender (male, female), race (white, black and others), and schooling (< 4 years, 4–8 years, > 8 years). The covariates related to TB included were: clinical form (PTB - pulmonary, EPTB - extra pulmonary, pulmonary + extra pulmonary), X-ray suspicious for TB (no, yes) and result of initial sputum smear (positive and negative). The genotype variables were those based on spoligotyping (LAM, non-LAM) and RD^Rio^ status (RD^Rio^, non-RD^Rio^).

### Statistical analysis

In order to identify risk factors for clustering, we performed univariate analysis using t-test for continuous variables and chi-square or the Fisher’s exact test for categorical variables. Factors that were significantly associated with clustering were analyzed by a multiple logistic regression test by a stepwise approach to identify factors that were independent predictors of clustering. We analyzed the isolates’ distribution according to cluster size and observed that the sample was not normally distributed. Thus, we defined the “cluster size” into four categories (clusters with 2–5, 6–9 or ≥10 isolates/cluster and unique patterns). Descriptive analysis of molecular and epidemiologic data was performed, according to cluster size classification. Based on a theoretical model for the study of determining TB [23], we performed crude analyses and we built one hierarchical polytomous regression model to identify factors associated with cluster size. Despite the categories of cluster size to imply an order, they did not meet the assumptions of an ordered logistic regression. Thus, we chose polytomous regression, which allows us to model simultaneously these multiple categories without the order assumption.

In the model we included all isolates analyzed. Unique pattern was defined as the reference group and was compared with the three cluster size categories.

The hierarchical levels for both models were defined as follows: level 1: the molecular variables (spoligotype and RD^Rio^ Genotype); level 2: the variables of level 1 and demographic variables (municipality of residence); level 3: the variables of level 2 and socio-demographic variables (age, gender, skin color and schooling); and level 4: the variables of level 3 and clinical variables (X-ray suspicious for TB, result of initial sputum smear, and TB clinical form). Therefore, the total effect of each variable is adjusted for the variables at the same level and the levels above. Descriptive data were shown as absolute and relative frequencies or mean value and standard deviation. Results from association analysis were presented as odds ratios (OR) with confidence intervals of 95% (95% CI). All analyses were conducted with the Stata® statistical package, version 13.0 (StataCorp LP, College Station, TX, USA).

## Results

Between January 2000 and December 2010, 5470 TB patients were diagnosed in the metropolitan area of Vitoria. Among these, 1320 (24%) had culture performed, and we obtained good quality RFLP patterns from 959 (72.6%) of them.

The IS*6110* RFLP analysis demonstrated that 461 (48%) cases had an isolate that belonged to a cluster and 498 (52%) had a unique pattern (Table 1). Cluster size ranged from two to 34 isolates and 108 (11.2%) formed a cluster with 10 or more isolates, 87 (9.1%) with 6–9 isolates and 266 (27.7%) with 2–5 isolates.

**Table 1:** Distribution of characteristics of TB patients according to their *M. tuberculosis* isolates’ IS6110 RFLP cluster status

Characteristics	Categories	All patients		Cluster		Unique Patterns (PU)		Odds Ratio
		*N* = 959	(%)	*N* = 461	(%)	*N* = 498	(%)	(95% CI)^d^
**Demographic**								
Age (years old)	< 20 years	100	(11.0)	62	(14.4)	38	(8.0)	1
	21–30 years	247	(27.3)	115	(26.7)	132	(27.9)	**0.53 (0.33–0.85)**
	31–40 years	220	(24.4)	100	(23.2)	120	(25.4)	**0.51 (0.31–0.82)**
	41–50 years	194	(21.5)	93	(21.6)	101	(21.4)	**0.56 (0.34–0.92)**
	> 50 years	143	(15.8)	61	(14.1)	82	(17.3)	**0.45 (0.27–0.76)**
Gender	Female	287	(30.0)	134	(29.0)	153	(31.0)	1
	Male	672	(70.0)	327	(71.0)	345	(69.0)	1.08 (0.82–1.42)
Race	White	190	(30.0)	90	(29.0)	100	(31.0)	1
	Black	119	(19.0)	64	(20.0)	55	(17.0)	1.29 (0.81–2.04)
	Others^a^	323	(51.0)	158	(51.0)	165	(52.0)	1.06 (0.74–1.52)
Schooling (years)	≤4 years	106	(21.0)	50	(21.0)	56	(22.0)	1
	5 to 8 years	304	(61.0)	150	(62.0)	154	(59.0)	1.09 (0.70–1.69)
	≥ 8 years	90	(18.0)	41	(17.0)	49	(19.0)	0.93 (0.53–1.64)
Metropolitan Area	Vitoria	583	(61.0)	290	(63.0)	293	(59.0)	1
	Vila Velha	89	(9.0)	40	(9.0)	49	(10.0)	0.82 (0.52–1.29)
	Cariacica	120	(13.0)	60	(13.0)	60	(12.0)	1.01 (0.68–1.49)
	Serra	123	(13.0)	53	(11.0)	70	(14.0)	0.76 (0.51–1.13)
	Outros	44	(5.0)	18	(4.0)	26	(5.0)	0.70 (0.37–1.30)
**Clinical**								
X-Ray	No	46	(6.0)	26	(8.0)	20	(5.0)	1
	Yes	667	(94.0)	318	(92.0)	349	(95.0)	0.99 (0.96–1.03)
TB Presentation	PTB^b^	779	(81.0)	390	(85.0)	389	(78.0)	1
	EPTB	111	(12.0)	40	(9.0)	71	(14.0)	**0.56 (0.37–0.84)**
	PTB + EPTB	69	(7.0)	31	(7.0)	38	(8.0)	0.81 (0.49–1.33)
AFB Smears Results	Negative	147	(15.0)	65	(14.0)	82	(17.0)	1
	Positive^c^	802	(85.0)	389	(86.0)	41 3	(83.0)	1.18 (0.83–1.69)
**Molecular**								
Spoligotyping	Non LAM	412	(48.0)	195	(50.0)	217	(46.0)	1
	LAM	448	(52.0)	193	(50.0)	255	(54.0)	0.84 (0.64–1.10)
RD^Rio^ Genotype	Non - RD^Rio^	552	(60.0)	253	(58.0)	299	(62.0)	1
	RD^Rio^	369	(40.0)	185	(42.0)	184	(38.0)	1.07 (0.99–1.15)

^a^ – Based on the SINAN response field for skin color

^b^– PTB = pulmonary TB, EPTB = extrapulmonary TB

^c^- All smear positive results were stratified as + 1 (10–99 AFB/100 fields), + 2 (1–10 AFB/field) and + 3 (> AFB/field)

^d^ – Crude odds ratio from univariate logistic regression. CI, confidence interval

All clusters were grouped into 30 RFLP families and six of these comprised 24.1% of the clustered isolates, while 108 (11.2%) belonged to the six largest clusters (≥ 10 isolates).

**Fig. 1:**
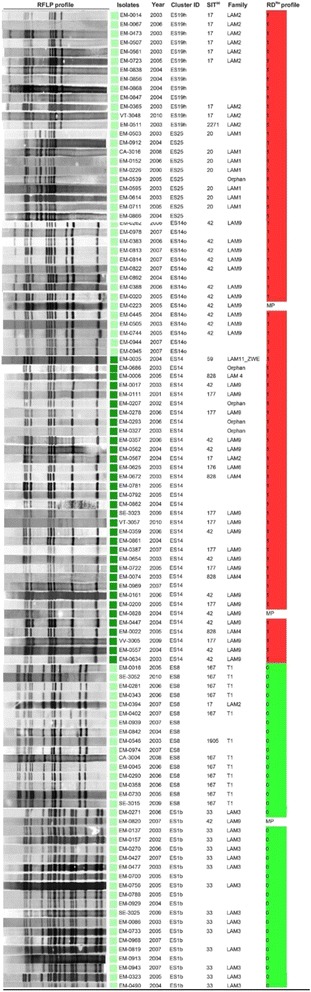
Genotypic Profile of the six largest clusters found in the study. (a) – SIT (International Shared Type), (b) - RD^Rio^ profile - (0): not RD^Rio^, (1): RD^Rio^ and (MP): mixed population

The cluster with the highest number of isolates was ES14 containing 34 followed by ES1b, ES8, ES14o, ES19h and ES25, which included 20, 16, 15, 13 and 10 isolates, respectively (Fig. 1). The ES14 genotype has an eight band pattern and is a member of the largest family (*n* = 86), sharing this pattern with one to three additional bands. In addition, the ES14 cluster has been present throughout the 11-year period and in 2003, 68% of all TB cases belonging to the largest clusters were caused by three clonal groups ES14, ES19h and ES25. However, in 2007, the clusters ES14o, ES1b and ES8 contributed to 88% of all TB cases belonging to larger clusters.

We found that all isolates of the ES14 cluster were of the RD^Rio^ genotype while the other large clusters ES1b (20 isolates) and ES8 (16 isolates) were exclusively non-RD^Rio^ (WT).

Of the total isolates spoligotyped, 448 (52%) were classified as LAM and 412 (48%) as non-LAM. Spoligotyping analysis for ES14 family showed one predominant sublineage (LAM9/SIT42; *n* = 42 [42/86 = 49%].

The results of univariate analysis of variables with genotype clustering status are presented in Table 1. Patients with extrapulmonary TB (EPTB) group were less likely to be infected with a cluster strain than those with pulmonary TB (PTB) (OR = 0.56, 95% CI 0.37–0.84; OR = 0.98, 95% CI 0.98–0.99, respectively). Individuals aged 21 to 30 years, 31 to 40 years, 41–50 years and > 50 years were less likely to be infected with a cluster strain than compared to individuals < 20 years of age (OR = 0.53, 95% CI 0.33–0.85, OR = 0.51 95% CI 0.31–0.82, OR = 0.56, 95% CI 0.34–0.92 and OR = 0.45, 95% CI 0.27–0.76).

Table 2 summarizes the demographic, clinical characteristics and laboratory findings of TB patients with isolates belonging to a cluster (2–5, 6–9 and ≥10) or a unique pattern genotype.

**Table 2:** Distribution of characteristics of TB patients according to their *M. tuberculosis* isolates’ IS6110 RFLP cluster size

						cluster size					
Characteristics	Categories	All patients		PU^a^		2–5		6–9		≥ 10	
		N = 959	(%)	N = 498	(%)	*N* = 266	(%)	*N* = 87	(%)	*N* = 108	(%)
**Demographic**											
Age (years old)	< 20 years	100	(11.0)	38	(8.0)	36	(14.6)	17	(19.8)	9	(9.1)
	21–30 years	247	(27.3)	132	(27.9)	60	(24.4)	25	(29.1)	30	(30.3)
	31–40 years	220	(24.4)	120	(25.4)	50	(20.3)	23	(26.7)	27	(27.3)
	41–50 years	194	(21.4)	101	(21.4)	60	(24.4)	14	(16.3)	19	(19.2)
	> 50 years	143	(15.8)	82	(17.4)	40	(16.3)	7	(8.2)	14	(14.4)
Gender	Female	287	(30.0)	153	(31.0)	81	(30.0)	24	(28.0)	29	(27.0)
	Male	672	(70.0)	345	(69.0)	185	(70.0)	63	(72.0)	79	(73.0)
Race	White	190	(30.0)	100	(31.0)	56	(32.0)	20	(31.0)	14	(19.0)
	Black	119	(19.0)	55	(17.0)	30	(17.0)	14	(22.0)	20	(27.0)
	Others^b^	323	(51.0)	165	(52.0)	89	(51.0)	30	(47.0)	39	(53.0)
School Level (years)	≤ 4 years	106	(21.0)	56	(22.0)	32	(23.0)	10	(20.0)	8	(16.0)
	5–8 years	304	(61.0)	154	(59.0)	86	(61.0)	30	(61.0)	34	(67.0)
	≥ 8 years	90	(18.0)	49	(19.0)	23	(16.0)	9	(18.0)	9	(18.0)
Metropolitan Area	Vitória	583	(61.0)	293	(59.0)	162	(61.0)	63	(72.0)	65	(60.0)
	Vila Velha	89	(9.0)	49	(10.0)	25	(9.0)	7	(8.0)	8	(7.0)
	Cariacica	120	(13.0)	60	(12.0)	34	(13.0)	9	(10.0)	17	(16.0)
	Serra	123	(13.0)	70	(14.0)	36	(14.0)	4	(5.0)	13	(12.0)
	Others	44	(5.0)	26	(5.0)	9	(3.0)	4	(5.0)	5	(5.0)
**Clinical**											
X Ray	No	46	(6.0)	20	(5.0)	17	(9.0)	3	(4.0)	6	(7.0)
	Yes	667	(94.0)	349	(95.0)	178	(91.0)	65	(96.0)	75	(93.0)
TB Presentation	PTB^c^	779	(81.0)	389	(78.0)	217	(82.0)	79	(91.0)	94	(87.0)
	EPTB	111	(12.0)	71	(14.0)	26	(10.0)	6	(7.0)	8	(7.0)
	PTB + EPTB	69	(7.0)	38	(8.0)	23	(9.0)	2	(2.0)	6	(6.0)
AFB Smears Results	Negative	147	(15.0)	82	(17.0)	48	(18.0)	6	(7.0)	11	(10.0)
	Positive^d^	802	(85.0)	413	(83.0)	214	(82.0)	81	(93.0)	94	(90.0)
**Molecular**											
Spoligotyping	Non LAM	412	(48.0)	217	(46.0)	144	(61.0)	33	(47.0)	18	(22.0)
	LAM	448	(52.0)	255	(54.0)	91	(39.0)	37	(53.0)	65	(78.0)
RD^Rio^ Genotype	Non RD^Rio^	552	(60.0)	299	(62.0)	182	(70.0)	36	(50.0)	35	(33.0)
	RD^Rio^	369	(40.0)	184	(38.0)	78	(30.0)	36	(50.0)	71	(77.0)

^a^ – When the unique patterns (PU) were the reference group and were compared with all others genotyped TB cases by

cluster size in number of patients per cluster

^b^ – Based on the SINAN response field for skin colour

^c^– PTB = pulmonary TB, EPTB = extrapulmonary TB

^d^- All smear positive results were stratified as + 1 (10–99 AFB/100 fields), + 2 (1–10 AFB/field) and + 3 (> AFB/field)

The hierarchical polytomous regression model (Table 3) showed that at the first level, those patients in the 6–9 and with ≥10 isolates/cluster group were more likely to belong to the LAM lineage (adjusted OR = 1.17, 95% CI 1.08–1.26; adjusted OR = 1.25, 95% CI 1.14–1.37, respectively), using unique patterns as the reference.

**Table 3:** Crude and adjusted odds ratio by hierarchical polytomous regression analysis of the association of characteristics of TB patients according to their *M. tuberculosis* isolates’ IS6110 RFLP cluster patterns (unique patterns for reference)

	Crude OR			Adjustment OR		
	Cluster Size			Cluster Size		
	2–5	6–9	≥ 10	2–5	6–9	≥ 10
	OR (CI)	OR (CI)	OR (CI)	OR (CI)	OR (CI)	OR (CI)
Level 1						
Spoligotyping						
Non LAM	Ref	Ref	Ref	Ref	Ref	Ref
LAM	1.08 (1.01–1.15)	1.18 (1.09–1.27)	1.22 (1.14–1.31)	1.08 (1.01–1.15)	1.17 (1.08–1.26)	1.22 (1.14–1.31)
RD^Rio^ Genotype						
Non-RD^Rio^	Ref	Ref	Ref	Ref	Ref	Ref
RD^Rio^	0.91 (0.81–1.03)	1.26 (1.15–1.38)	1.06 (0.94–1.19)	0.91 (0.80–1.03)	1.25 (1.14–1.37)	1.05 (0.93–1.18)
Level 2						
Metropolitan Area						
Vitoria	Ref	Ref	Ref	Ref	Ref	Ref
Vila Velha	0.92 (0.55–1.55)	0.66 (0.28–1.53)	0.73 (0.33–1.62)	1.00 (0.59–1.69)	0.74 (0.30–1.77)	1.04 (0.46–2.36)
Cariacica	1.02 (0.64–1.62)	0.70 (0.32–1.47)	1.27 (0.69–2.33)	1.12 (0.70–1.80)	0.71 (0.32–1.58)	1.81 (0.96–3.40)
Serra	0.93 (0.59–1.45)	0.26 (0.09–0.75)	0.83 (0.43–1.60)	0.99 (0.63–1.55)	0.29 (0.10–0.84)	1.08 (0.55–2.11)
Outros	0.62 (0.28–1.36)	0.71 (0.24–2.12)	0.86 (0.32–2.34)	0.66 (0.30–1.45)	0.74 (0.24–2.28)	1.04 (0.37–2.89)
Level 3						
Gender						
Female	Ref	Ref	Ref	Ref	Ref	Ref
Male	1.01 (0.73–1.40)	1.16 (0.70–1.93)	1.20 (0.75–1.92)	0.98 (0.70–1.39)	1.19 (0.69–2.05)	1.20 (0.72–2.00)
Age (years old)						
< 20 years	Ref	Ref	Ref	Ref	Ref	Ref
21–30 years	0.48 (0.28–0.83)	0.42 (0.21–0.86)	0.96 (0.42–2.19)	0.49 (0.28–0.85)	0.45 (0.21–0.97)	1.13 (0.47–2.69)
31–40 years	0.44 (0.25–0.77)	0.43 (0.21–0.88)	0.95 (0.41–2.19)	0.43 (0.24–0.77)	0.41 (0.19–0.90)	1.01 (0.42–2.43)
41–50 years	0.62 (0.36–1.09)	0.31 (0.14–0.69)	0.79 (0.33–1.90)	0.61 (0.34–1.08)	0.30 (0.12–0.70)	0.93 (0.37–2.34)
> 50 years	0.51 (0.28–0.93)	0.19 (0.07–0.50)	0.72 (0.28–1.81)	0.49 (0.26–0.91)	0.18 (0.06–0.49)	0.89 (0.33–2.37)
Race						
White	Ref	Ref	Ref	Ref	Ref	Ref
Black	0.97 (0.56–1.69)	1.27 (0.59–2.71)	2.59 (1.21–5.54)	0.82 (0.46–1.46)	0.79 (0.351.81)	2.02 (0.90–4.51)
Others	0.96 (0.63–1.46)	0.90 (0.49–1.68)	1.68 (0.87–3.26)	0.89 (0.58–1.38)	0.76 (0.39–1.49)	1.53 (0.77–3.05)
School level, years						
≤ 4 years	Ref	Ref	Ref	Ref	Ref	Ref
4 to 8 years	0.97 (0.58–1.62)	1.09 (0.50–2.37)	1.54 (0.67–3.53)	0.89 (0.52–1.51)	1.01 (0.42–2.41)	1.49 (0.62–3.57)
≥ 8 years	0.82 (0.42–1.58)	1.02 (0.38–2.73)	1.28 (0.46–3.58)	0.63 (0.31–1.27)	0.70 (0.24–2.12)	1.23 (0.41–3.70)
Level 4						
X-ray						
Negative	Ref	Ref	Ref	Ref	Ref	Ref
Suspicious of TB	0.60 (0.30–1.17)	1.24 (0.35–4.29)	0.71 (0.27–1.84)	0.34 (0.14–0.79)	0.35 (0.08–1.56)	0.27 (0.08–0.92)
Sputum smear results						
Negative	Ref	Ref	Ref	Ref	Ref	Ref
Positive	0.88 (0.60–1.31)	2.68 (1.13–6.34)	1.69 (0.87–3.30)	0.69 (0.41–1.17)	2.05 (0.68–6.16)	1.24 (0.53–2.90)
TB presentation						
PTB	Ref	Ref	Ref	Ref	Ref	Ref
EPTB	0.65 (0.40–1.05)	0.41 (0.17–0.99)	0.46 (0.21–1.00)	0.44 (0.23–0.83)	0.41 (0.13–1.21)	0.37 (0.13–1.03)
PTB + EPTB	1.08 (0.63–1.86)	0.26 (0.06–1.09)	0.65 (0.27–1.60)	0.94 (0.49–1.78)	0.29 (0.06–1.34)	0.68 (0.23–1.98)

*OR* odds ratio, *CI* confidence interval, *SD* standard deviation, *TB* tuberculosis, *PTB* pulmonary TB, *EPTB* extrapulmonary TB. ^*^Hierarchical levels: Level 1 = Spoligotyping + RD^Rio^ genotype; Level 2 = Spoligotyping + RD^Rio^ genotype + Metropolitan Area; Level 3 = Spoligotyping + RD^Rio^ genotype + Metropolitan Area + Gender +Age + Race + Schooling; Level  4 = Spoligotyping + RD^Rio^ genotype + Metropolitan Area + Gender +Age + Race + Schooling + X-Ray + Sputum smear results + TB presentation

On the other hand, subjects in the 2–5 isolates/cluster group were less likely to belong to the RD^Rio^ genotype (adjusted OR = 0.91, 95% CI 0.80–1.03). At the second level, living in a specific neighborhood (Serra city) in the metropolitan area of Vitória decrease the risk of being in the 6–9 isolates/cluster group (adjusted OR = 0.29, 95% CI, 0.10–0.84), using unique patterns as the reference group. In addition, at the third level individuals aged 21 to 30 years, 31 to 40 years and > 50 years were less likely of belonging the 2–5 isolates/cluster group than unique patterns compared to individuals < 20 years of age (adjusted OR = 0.49, 95% CI 0.28–0.85, OR = 0.43 95% CI 0.24–0.77 and OR = 0.49, 95% CI 0.26–0.91) respectively.

Suspicion of TB on chest x-ray was less likely observed for those infected with strains in the 2–5 isolates/cluster group (adjusted OR = 0.35, 95% CI 0.15–0.79) than unique patterns. Interestingly, the extrapulmonary disease was less likely to occur in those infected with strains in the 2–5 isolates/cluster group (adjustment OR = 0.45, 95% CI 0.24–0.85) than unique patterns. Furthermore, TB patients whose isolates were included in the 6–9 isolates/cluster group (crude OR = 2.68, 95% IC 1.13–6.34) were more likely to be smear-positive compared to unique patterns as the reference. The difference was not statistically significant, however, after regression analysis.

## Discussion

Since two decades ago, many studies on transmission of TB have been complemented by genotyping techniques. The IS6110 RFLP test has been used to distinguish patients with TB due to recent transmission from reactivation disease [24]. In the present study, we evaluated transmission dynamics of TB in Vitoria, Brazil, during a 11-year period, comparing demographic, clinical and epidemiologic characteristics with Mtb genotypes and genotype clustering. We observed that a large proportion of recently transmitted TB was due to a limited set of Mtb genotypes and that certain cluster sizes were associated with patient demographic, clinical, or epidemiological characteristics.

A limitation of our study is that our patient data were derived from the SINAN secondary database with limited data. Data such as smoking, HIV status, drug abuse and drug susceptibilities testing, at the time of the study, are not regularly reported by SINAN. On the other hand, the same database was the basis for studies on disease surveillance as described in earlier studies [11, 22, 25, 26]. The strength of the study is the large sample size, offering a statistical power that is higher than in most other studies. In addition, the long study period increases the chance of finding epidemiological links through genotyping of the Mtb strains, while that a small sample size and a poorly defined area can underestimate clustering proportions [27].

We provide evidence that six Mtb (cluster) strains have consistently contributed to the high burden of recent-transmission TB from 2000 to 2011 in the Metropolitan area of Vitória-ES. They accounted for 12% of all culture-confirmed TB cases in this area during this period. This observation was possible probably because of the high case coverage and length of the study period, increasing the chance of genotype clusters, as reported by van Soolingen et al. [28]. This finding is in concordance with a failing TB control program that fails to stop disease transmission in this area, and better approach should be implemented. This may be related to a variety of factors including delay in diagnosis and poor contact investigation strategies. Maciel and colleagues recently suggested a possibility for implementing a new case-finding strategy based on screening populations in neighborhoods with high-density recent-transmission TB and social network analyses [29].

In the present study, we found that isolates of the ES14 family accounted for the largest proportion of recently-transmitted TB cases, which suggests that these strains are either more transmissible or more likely to cause disease after infection. Strains of this family were LAM (mostly LAM9) family and RD^Rio^ genotype. Isolates with the particular eight band pattern that is the basis of this family have been reported as predominant also in studies conducted in Rio de Janeiro, São Paulo and Rio Grande do Sul in Brazil, and is frequently encountered in a database of isolates originating from other countries such as the Caribbean, Europe, Africa and other countries in South America [21, 30–32]. These findings suggest that the incidence of TB in this region may be strongly influenced by a relatively small subset of actively circulating strains. It is known that in areas with a higher incidence of TB, RFLP patterns are often less variable than in low-incidence areas [33]. Recently Ribeiro et al. (2015) showed that new TB cases do not just cluster in space, but that certain *M. tuberculosis* lineages tend to cluster even after controlling for known individual and socioeconomic factors that can influence transmission [29].

In our study, the clinical manifestation of TB—pulmonary or extrapulmonary TB—was associated with any particular cluster size (2–5 isolates/cluster). Although Gomes and colleagues showed no association between the clinical manifestation of TB and clustering rates, interestingly when stratified by cluster size a larger proportion of EPTB cases were in particular cluster size [25]. Our earlier studies also demonstrated that RD^Rio^ strains are less likely to cause extrapulmonary disease than non-RD^Rio^ strains [11] and the high prevalence of RD^Rio^ strains in the present study could be associated with levels of clustering of EPTB cases but many other factors influence clinical manifestations of TB, including the duration of illness before diagnosis as well as underlying host factors.

Our findings are consistent with several studies which have demonstrated the predominance of isolates of the LAM family and of the RD^Rio^ lineage in TB cases in Brazil [9, 11, 34–36]. *Lazzarini* and colleagues showed that the LAM1 and LAM2 sublineages exclusively belonged to the RD^Rio^ genotype, while the lineages LAM4, LAM5, LAM6 and LAM9 included both RD^Rio^ and non RD^Rio^ genotypes, and LAM3 were all non-RD^Rio^ [9], although some exceptions on this rule were presented recently [32]. Indeed, previous studies showed that RD^Rio^ genotype is significantly associated with cluster groups (an indication of recent transmission) than non-RD^Rio^ strains, both in Brazilians and in non-Brazilian populations [36]. These data corroborate with findings in our study that showed that isolates from RD^Rio^ genotype belong to 6–9 isolates/cluster group. Although the proportion of cases with more 6–9 isolates/cluster patterns among RD^Rio^ strains was significantly greater than that non RD^Rio^ strains, it is not clear if this difference could be attributed to enhanced virulence and transmissibility of the RD^Rio^ strains. Previous studies suggested that these strains were recently introduced in some regions of Brazil and evolved after its introduction, or that the RD^Rio^ strains are more biologically “fit” [11, 36].

Glynn and colleagues suggested that these cluster strains are particularly transmissible or particularly more likely to cause disease after infection [36]. Other possibilities for their predominance are that they have been present in a geographic setting longer than others and that they had more time to become widespread, or that we are seeing a founder effect in some populations with subsequent spread following human migration patterns [33].

## Conclusions

Our findings suggest that strains belonging to the LAM family and RD^Rio^ genotype showed are more likely to be largest clusters (6–9 and ≥10 isolates/cluster). We confirmed that the ES14 family is the most prevalent genotype of Mtb in Vitória – ES, Brazil, this suggesting, a large proportion of TB cases in one city can be caused by a few set of lineages circulating in the city. This provides an opportunity to characterize factors that affect transmission instead of host factors. Therefore, once an *M. tuberculosis* lineage enters in a community, that particular strain and its related family strains are more likely to propagate than outside strains.

## Abbreviations

AFB: Acid-Fast Bacilli
CI: Confidence interval
DATASUS: Department of Brazilian Ministry of Health
DNA: Deoxyribonucleic Acid
EPTB: Extrapulmonary TB
ES: abbreviation for clusters in Espirito Santo
IS*6110*: Insertion sequence *6110*
LAM: Latin American Mediterranean
LSP: Long Sequence Polymorphism
Mtb: *Mycobacterium tuberculosis*
MTBC: Mycobacterium tuberculosis complex
NDI/UFES: Núcleo de Doenças Infecciosas at the Federal University of Espirito Santo
OR: Odds ratio
PCR: Polymerase Chain Reaction
PTB: Pulmonary TB
*Pvu* II: restriction endonuclease type II
RD: Regions Differences
RFLP: Restriction fragment length polymorphism
SINAN: Brazilian national surveillance system
TB: Tuberculosis
UPGMA: Unweighted pair group method of arithmetic average
